# Reactive Oxygen Species Differentially Modulate the Metabolic and Transcriptomic Response of Endothelial Cells

**DOI:** 10.3390/antiox11020434

**Published:** 2022-02-21

**Authors:** Niklas Müller, Timothy Warwick, Kurt Noack, Pedro Felipe Malacarne, Arthur J. L. Cooper, Norbert Weissmann, Katrin Schröder, Ralf P. Brandes, Flávia Rezende

**Affiliations:** 1 Institute for Cardiovascular Physiology, Goethe University, Theodor-Stern Kai 7, 60590 Frankfurt, Germany; nmueller@vrc.uni-frankfurt.de (N.M.); warwick@vrc.uni-frankfurt.de (T.W.); kunoack@students.uni-mainz.de (K.N.); malacarne@vrc.uni-frankfurt.de (P.F.M.); schroeder@vrc.uni-frankfurt.de (K.S.); brandes@vrc.uni-frankfurt.de (R.P.B.); 2 German Center of Cardiovascular Research (DZHK), Partner Site Rhein Main, 60590 Frankfurt, Germany; 3 Department of Biochemistry and Molecular Biology, New York Medical College, 15 Dana Road, Valhalla, NY 10595, USA; arthur_cooper@nymc.edu; 4 Justus Excellence Cluster Cardio-Pulmonary Institute (CPI), University of Giessen and Marburg Lung Center (UGMLC), Member of the German Center for Lung Research (DZL), Justus-Liebig-University, 35390 Giessen, Germany; norbert.weissmann@innere.med.uni-giessen.de

**Keywords:** endothelial cells, metabolomics, RNAseq, reactive oxygen species, D-amino acid oxidase

## Abstract

Reactive oxygen species (ROS) are important mediators of both physiological and pathophysiological signal transduction in the cardiovascular system. The effects of ROS on cellular processes depend on the concentration, localization, and duration of exposure. Cellular stress response mechanisms have evolved to mitigate the negative effects of acute oxidative stress. In this study, we investigate the short-term and long-term metabolic and transcriptomic response of human umbilical vein endothelial cells (HUVEC) to different types and concentrations of ROS. To generate intracellular H_2_O_2_, we utilized a lentiviral chemogenetic approach for overexpression of human D-amino acid oxidase (DAO). DAO converts D-amino acids into their corresponding imino acids and H_2_O_2_. HUVEC stably overexpressing DAO (DAO-HUVEC) were exposed to D-alanine (3 mM), exogenous H_2_O_2_ (10 µM or 300 µM), or menadione (5 µM) for various timepoints and subjected to global untargeted metabolomics (LC-MS/MS) and RNAseq by MACE (Massive analysis of cDNA ends). A total of 300 µM H_2_O_2_ led to pronounced changes on both the metabolic and transcriptomic level. In particular, metabolites linked to redox homeostasis, energy-generating pathways, and nucleotide metabolism were significantly altered. Furthermore, 300 µM H_2_O_2_ affected genes related to the p53 pathway and cell cycle. In comparison, the effects of menadione and DAO-derived H_2_O_2_ mainly occurred at gene expression level. Collectively, all types of ROS led to subtle changes in the expression of ribosomal genes. Our results show that different types and concentration of ROS lead to a different metabolic and transcriptomic response in endothelial cells.

## 1. Introduction

The vascular system is entirely lined by a single layer of endothelial cells (EC), which facilitate the exchange of nutrients and oxygen between the blood and surrounding tissues. Angiogenic endothelial cells migrate into hypoxic tissue, and endothelial nitric oxide (NO) production inhibits complex IV of the respiratory chain. Endothelial ATP production therefore highly relies on glycolysis instead of oxidative phosphorylation. In turn, EC have a relative small mitochondrial volume [1,2]. The endothelial production of ROS is triggered by oxidants produced from activated immune cells, cytokines, or physical stimuli, such as oscillatory flow [3,4]. Since ROS can lead to oxidative stress, various anti-oxidant defense mechanisms have evolved, such as detoxifying enzymes, redox-sensitive gene expression, and a dynamic metabolic response.

In the context of redox signaling, ROS can be produced and act in a confined space in a compartmentalized and controlled manner [5]. It is well accepted that not only the concentration, but also the type of ROS determines the biological effects as oxidants such as H_2_O_2_ and superoxide have different chemical properties. H_2_O_2_ can selectively oxidize low pKa thiols and transition metals in proteins [6,7] whereas O_2_^•−^ reacts preferentially with iron sulfur clusters and nitric oxide [8,9,10,11,12]. In response to an acute ROS challenge, changes in metabolism can be expected to precede changes in gene expression. This is due to the fact that ROS-dependent inactivation of metabolic enzymes has immediate consequences on the concentration of the upstream and downstream metabolites of the affected enzyme. However, the metabolic and transcriptomic response of EC to different types and concentrations of ROS has not been studied in great detail. Appropriate tools to study the complex cellular response to intracellular oxidants, particularly over the course of time, have been lacking. Conclusions regarding the biological role of H_2_O_2_ in signaling are based on findings generated by the addition of H_2_O_2_ to cultured cells. However, this does not reflect the dynamics of intracellular H_2_O_2_ flux in the regulation of signaling events [13,14,15].

To compare the metabolic and transcriptomic effects of intracellularly generated H_2_O_2_ to other types of ROS, we utilized a chemogenetic approach based on overexpression of human D-amino acid oxidase (DAO). DAO oxidizes D-amino acids to their corresponding imino acids and H_2_O_2_. The imine is then non-enzymatically hydrolyzed to its corresponding α-keto acid [13,16,17,18]. The enzyme is stereospecific for D-amino acids (e.g., D-alanine) which allows for precise activation of the enzyme. This makes DAO a useful tool to study how the intracellular production of H_2_O_2_ modulates cellular response. With this tool in hand, we analyzed the different metabolic and transcriptomic responses of HUVEC to DAO-derived H_2_O_2_ in comparison to exposure to exogenous H_2_O_2_ (10 µM and 300 µM) or menadione (to generate intracellular O_2_^•−^) in a time dependent manner.

## 2. Materials and Methods

### 2.1. Chemicals

All chemicals were purchased from Sigma-Aldrich (St. Louis, MO, USA) unless otherwise indicated.

### 2.2. Cell Culture

Human umbilical vein endothelial cells (HUVEC) were purchased from Lonza (CC-2519, Lot No. 371074, 369146, 314457, 192485, 186864, 171772, Walkersville, MD, USA). HUVEC were cultured in endothelial growth medium supplemented with human recombinant epidermal growth factor (EGF), Endo-CGS-Heparin (PeloBiotech, Planegg, Germany), 8% fetal calf serum (FCS, #S0113, Biochrom, Berlin, Germany), penicillin (50 U/mL) and streptomycin (50 μg/mL) (#15140-122, Gibco/Lifetechnologies, Carlsbad, CA, USA). For each experiment, at least three different batches of HUVEC at passage 4 were used.

Human embryonic kidney 293 cells (HEK293) cells were obtained from ATCC (Manassas, VA, USA) and Lenti-X 293T cells for virus production were purchased from Takara (#632180, Takara, Japan). Both cell lines were cultured in Dulbecco’s Modified Eagle Medium High Glucose (DMEM High Glucose, Gibco, Carlsbad, CA, USA) supplemented with FCS (8%), penicillin (50 U/mL), and streptomycin (50 μg/mL) (#15140-122, Gibco/ Lifetechnologies, Carlsbad, CA, USA). All cells were cultured in a humidified atmosphere (5% CO_2_, 37 °C). 

### 2.3. Immunofluorescence

Cells were seeded on 8-well immunofluorescence plates (Ibidi, Gräfeling, Germany). At 80% confluence, cells were washed with PBS, fixed with 4% paraformaldehyde and permeabilized with 0.05% Triton X-100. After a blocking step with 3% BSA (bovine serum albumin) for 30 min, cells were incubated at 4 °C overnight with a 1:200 dilution of the primary antibody against human D-amino acid oxidase (#ab196563, abcam, Cambridge, UK). Cells were washed with 0.3% Tween 20 in PBS and incubated with a 1:500 dilution of the secondary antibody (Rabbit IgG (Alexa Fluor 647, #A31573, Invitrogen, Carlsbad, CA, USA) for 30 min. The cells were then washed and counterstained with 4′,6-diamidino-2-phenylindole (DAPI). Images were captured with a laser confocal microscope LSM800 (Zeiss, Jena, Germany) and analyzed with ZEN lite software (Zeiss, Jena, Germany).

### 2.4. Cloning of pLVX2 hDAO for Lentiviral Overexpression

The pLVX2-CIBN-GFP-CAAX PuroRes [19] vector was a gift from X. Trepat (Barcelona, Spain). Human DAO 10xHis-Tag was amplified from pCMV6-h-DAO (#HG13372-CH, Sino Biological Inc., Beijing, China) with the following primers: 5′- ACA CCT TCG AAA TGC GTG TGG TGG TG -3′ and 5′- ACA CCG CGG CCG CTT AGT GAT GGT GGT G-3′. Both the vector and the PCR product of hDAO, were digested with BstBI/NotI (#FD0124, #FD0593, ThermoFisher, Dreieich, Germany), purified by gel extraction, and ligated (#15224025, T4 DNA ligase, ThermoFisher, Dreieich, Germany) as per the manufacturer’s instructions. The final plasmid (pLVX2 hEF1α human DAO 10xHis-Tag) was purified and sequenced.

### 2.5. Lentiviral Transduction 

Pseudotyped lentiviruses were produced by transfecting LentiX cells with pLVX2- hDAO-10xHis-Tag together with lentiviral packaging plasmids (#12260 and #12259, Addgene, Watertown, MA, USA) as described previously [20] using PEI (polyethyleneimine). Viral supernatants were collected, filtered, and snap-frozen on the third day after transfection. HUVEC (but also HEK293 cells as control, HEK-DAO) were transduced with hDAO-10xHis-Tag viral particles for 24 h and then selected with puromycin (2 µg/mL) for 7–10 days.

### 2.6. Determination of ROS Production

ROS production was measured as described previously [21] using a luminol-based chemiluminescence assay. In brief, luminol (100 μM) horseradish peroxidase (HRP, 1 unit/mL) coupled chemiluminescence was measured in a Berthold 6-channel luminometer (LB9505, Berthold, Wildbad, Germany). All measurements were performed in HEPES-Tyrode buffer containing in mmol/L: 137 NaCl, 2.7 KCl, 0.5 MgCl_2_, 1.8 CaCl_2_, 5 glucose, 0.36 NaH_2_PO_4_, 10 HEPES. PEG-catalase (500 U/mL) was directly added to the sample during the measurement. 

H_2_O_2_ was also measured with the Amplex Red^®^/HRP (50 μm, Invitrogen, Carlsbad, USA; HRP, 2 units/mL) fluorimetric assay as previously described [7,22]. Fluorescence was determined in a microplate reader (excitation 530 nm, emission 590 nm) and normalized to the protein amount as determined by the Bradford protein assay. To calculate the catalase-sensitive portion of the signal, PEG-catalase (500 U/mL) was added to the assay buffer 30 min before starting the assay. 

### 2.7. Redox Western Blots

For peroxiredoxin western blots, cells were exposed to H_2_O_2_, D-alanine, or L-alanine or menadione for 10 min. Alternatively, cells were pre-incubated with the thioredoxin reductase inhibitor auranofin (3 µM, 20 min). Free thiols were blocked with N-ethylmaleimide (NEM, 100 mM). After a washing step with PBS-NEM (100 mM), cells were scraped in alkylation buffer (40 mM Hepes, 50 mM NaCl, 1 mM EGTA, Inhibitors, 100 mM NEM, pH 7.4) and 1% CHAPS (Applichem, Darmstadt, Germany) for solubilization. The protein concentration was determined by the Bradford assay. Samples were supplemented with non-reducing sample buffer (8.5% glycerol, 2% SDS, 6.25% TRIS/HCl pH 6.8, 0.013% bromophenol blue) and separated on an SDS-PAGE gel, followed by western blotting analysis. After incubation with primary antibodies, membranes were analyzed with an infrared-based detection system (LI-COR, Bad Homburg, Germany), using fluorescent-dye-conjugated secondary antibodies from LI-COR biosciences (Bad Homburg, Germany). The following antibodies were used: D-amino acid oxidase (DAO, #ab196563, Abcam, Cambridge, UK), peroxiredoxin 1 (Prx1, #MAB3488, R&D systems, Minneapolis, MN, USA), peroxiredoxin 2 (Prx2, #AF3489, R&D systems, Minneapolis, MN, USA), peroxiredoxin 3 (Prx3, #A304-744, Bethyl Laboratories, Montgomery, AL, USA), peroxiredoxin 4 (Prx4, #AF5460, R&D systems, Montgomery, AL, USA), and peroxiredoxin-SO_3_ (Prx-SO3, #ab16830, Abcam, Cambridge, UK).

### 2.8. Metabolomics 

HUVEC were grown in sister cultures that were treated identically. One dish was used for metabolic analysis while the corresponding sister culture was used to isolate the total RNA for RNASeq and data normalization. HUVEC at 80% confluence, were starved overnight in endothelial basal medium (PeloBiotech, Planegg, Germany) supplemented with 10 mM L-glutamine and 0.1% FCS. The next day, cells were exposed to H_2_O_2_ (10 μM or 300 μM), menadione (5 μM), D-alanine (3 mM), or basal medium (control sample) for 3, 10, 30, 90, 270, or 900 min. After exposure, cells were washed with ice-cold PBS and subsequently harvested in 80% LC/MS-grade methanol (Carl Roth, Karlsruhe, Germany) containing internal standards. Untargeted global metabolomics was performed by Metabolon Inc. (Morrisville, NC, USA) using a Waters ACQUITY ultra-performance liquid chromatography (UPLC) and a Thermo Scientific Q-Exactive high resolution/accurate mass spectrometer interfaced with a heated electrospray ionization (HESI-II) source and Orbitrap mass analyzer operating at 35,000 mass resolution as previously described [23,24,25]. Briefly, cell samples were extracted with methanol to remove the protein fraction. The extract was divided into five fractions: two for analysis by two separate reverse phase (RP)/UPLC-MS/MS methods with positive ion mode electrospray ionization (ESI), one for analysis by RP/UPLC-MS/MS with negative ion mode ESI, one for analysis by hydrophilic interaction chromatography (HILIC)/UPLC-MS/MS with negative ion mode ESI, and one sample as a backup. The extract was gradient eluted from a C18 column (Waters UPLC BEH C18-2.1 × 100 mm, 1.7 µm) using water and methanol, containing 0.05% perfluoropentanoic acid (PFPA) and 0.1% formic acid (FA). Another aliquot was also analyzed using acidic positive ion conditions, however, in this case, the method was chromatographically optimized for more hydrophobic compounds. In this method, the extract was gradient eluted from the same aforementioned C18 column using methanol, acetonitrile, water, 0.05% PFPA and 0.01% FA and was operated at an overall higher organic content. Another aliquot was analyzed using basic negative ion optimized conditions using a separate dedicated C18 column. The basic extracts were gradient eluted from the column using methanol and water, however with 6.5 mM ammonium bicarbonate at pH 8. The fourth aliquot was analyzed via negative ionization following elution from an HILIC column (Waters UPLC BEH Amide 2.1 mm × 150 mm, 1.7 µm) using a gradient consisting of water containing acetonitrile with 10 mM ammonium formate, pH 10.8. The MS analysis alternated between MS and data-dependent MS^n^ scans using dynamic exclusion. The scan range varied slighted between methods but covered 70–1000 m/z. Raw data were extracted, peak-identified, and quality control-processed by Metabolon [26]. Compounds were identified by comparison to library entries with over 3300 commercially available purified standard compounds [24]. A batch correction was performed by Metabolon. Following log transformation and imputation of missing values, if any, with the minimum observed value for each compound, Mixed Model Contrasts were used to identify biochemicals that differed significantly between experimental groups. In parallel, metabolomic results were normalized to the RNA values of the corresponding sister culture, missing values were imputed, and statistically analyzed using log transformed data. *p* values of <0.05 were considered significant. Visualizations and plots of metabolomics data were generated using the ggplot (3.3.5) package in R (4.1.1). Each treatment was analyzed with respect to its control.

### 2.9. RNA Isolation and RNAseq by Massive Analysis of cDNA Ends (MACE)

RNA of sister cultures was isolated with the RNA Mini Kit from (Bio&Sell, Nuremberg, Germany) combined with on-column DNase digestion (DNase-Free DNase Set, Qiagen, Hilden, Germany) to avoid contamination by genomic DNA. The libraries were prepared using GenXPro MACE kit v.2.0. RNA was sheared to an average size of 350 bps followed by poly-A specific cDNA synthesis. The PCR product was purified by SPRI (solid phase reversible immobilization) purification and the final product was quality controlled on a PerkinElmer LabChip GXII. The fragments were ligated to “TrueQuant” (GenXPro property, containing unique molecule identifiers). This unique identifier helps to remove PCR duplicates. MACE-tags were amplified with 10 PCR cycles and the libraries were sequenced on an Illumina NextSeq 500 machine. MACE sequencing reads for all samples were quantified against the *hg38* transcriptome (obtained from *Ensembl)* [27,28]. Reads not aligned to the transcriptome were discarded at this point. Differential gene expression analysis was performed using *DESeq2* (1.32.0) [29]. Raw transcript counts were summed per gene and used in the standard *DESeq2* differential gene expression analysis workflow, using a negative binomial test over gene counts in each of the combinations of conditions. Differences in gene expression (Differentially expressed genes, DEG) between conditions were considered significant with an adjusted (Benjamini-Hochberg) *p* value < 0.05. Differentially expressed genes per exposure type were subjected to pathway enrichment analysis in *R* using *ClusterProfiler* (4.0.5) and the *enrichKEGG* function [30]. Significantly enriched pathways versus a default random background gene set were those with an adjusted (Benjamini-Hochberg) *p* value < 0.05. Gene heatmaps were hierarchically clustered in *R* using *hclust* with Euclidean distances and the *ward.D* method. 

### 2.10. Gene Correlation and Transcription Factor Analysis

Transcription factor (TF) analysis from differentially expressed genes (DEGs) was performed using enrichR (3.0) with TFs enriched in the “ENCODE and ChEA consensus TFs from ChiP-X” database. Enriched TFs were considered significant with an adjusted (Benjamini-Hochberg) *p* value < 0.05. Correlations were performed using a Pearson correlation test in *R*. Visualizations and plots were generated using *ggplot2* (3.3.5) [31].

### 2.11. Statistics

Unless otherwise indicated, data are shown as means ± standard error of the mean (SEM). Calculations were performed with Prism 9.0 or R (4.1.1). For multiple group comparisons, ANOVA followed by Bonferroni or Benjamini Hochberg post hoc testing was performed. A *p*-value of <0.05 was considered as significant. n indicates the number of individual experiments. No samples were excluded from the analysis.

## 3. Results

### 3.1. DAO Is an Efficient Chemogenetic Tool to Produce H_2_O_2_ Intracellularly 

**Figure 1:**
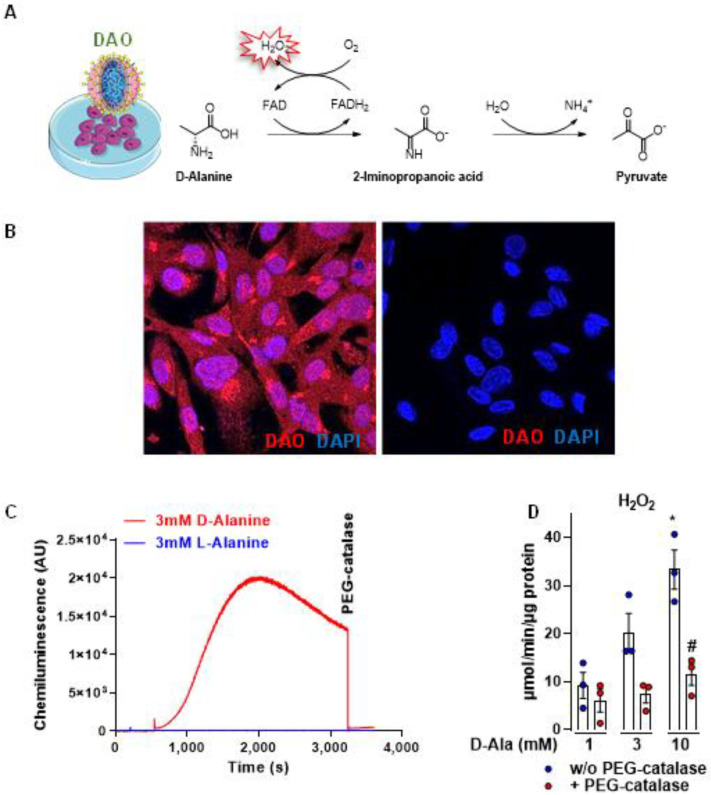
Generation and characterization of DAO as a chemogenetic approach to study the met-abolic and transcriptomic response to intracellular H_2_O_2_ in HUVEC. (**A**) Lentiviral overexpression of human D-amino acid oxidase (DAO) and its chemical reaction. (**B**) Immuno-fluorescence for DAO in HUVEC-DAO (**left**) and HUVEC-CTL (empty vector, **right**). (**C**) H_2_O_2_ measurements in HUVEC using Luminol/HRP and (**D**) Amplex red®/HRP assay * *p* < 0.05 10 mM D-Ala versus 1 mM D-Ala; # *p* < 0.05 10 mM D-Ala plus vs 10 mM D-Ala minus PEG-catalase. One-way-ANOVA with Bonferroni correction.

We cloned human DAO into a lentiviral plasmid to overexpress the enzyme in HUVEC to generate intracellular H_2_O_2_ upon stimulation with D-alanine (Figure 1A,B). DAO expression after transduction was readily detected in HUVEC by immunofluorescence. To test the activity of DAO, H_2_O_2_ was measured by luminol/ HRP chemiluminescence. Addition of 3 mM D-Ala resulted in a strong increase in chemiluminescence (Figure 1C). Likewise, with the Amplex red^®^/HRP assay, increasing concentrations of D-alanine (1–10 mM), but not L-alanine, resulted in an increased H_2_O_2_ production. PEG-catalase reduced the detected level of H_2_O_2_ (Figure 1D) whereas the DAO inhibitor 4HF (4H-furo [3,2-b]pyrrole-5-carboxylic acid, 1 µM) almost entirely abolished the D-alanine-elicited H_2_O_2_ production (Supplementary Figure S1). Thus, the HUVEC-DAO system is a valid tool to increase cellular H_2_O_2_ level in a D-alanine-dependent manner.

### 3.2. Different Types of ROS Elicit a Differential Metabolic Response in HUVEC

**Figure 2:**
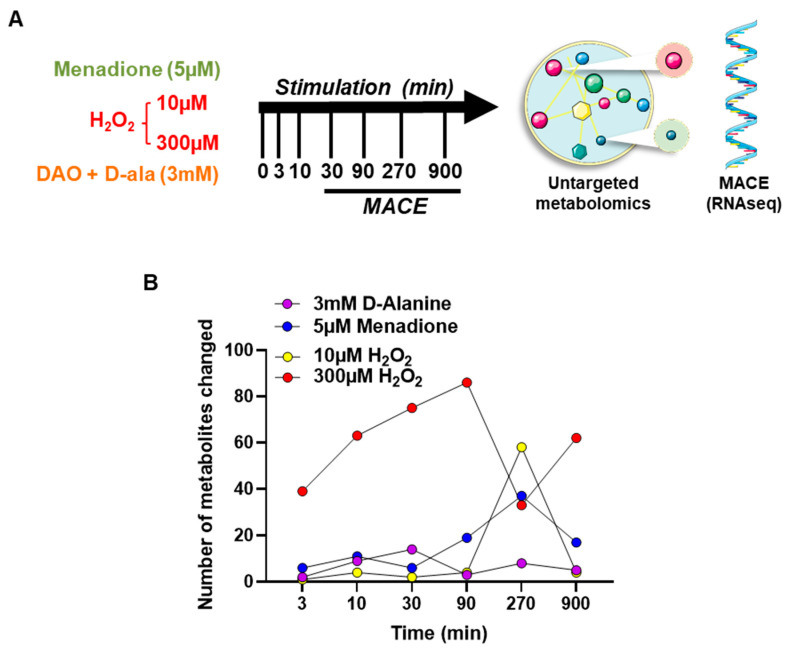
Time course analysis for metabolomics and transcriptomics of HUVEC with different oxidative stimuli. (**A**): Experimental design. (**B**): Number of metabolites significantly altered upon exposure to different ROS in HUVEC (*n* = 3).

We designed a large-scale experiment to determine the short-term and long-term metabolic and transcriptomic response of HUVEC to different types and concentrations of ROS (Figure 2A). 

HUVEC-DAO were stimulated with 3 mM D-alanine to generate H_2_O_2_ intracellularly. In comparison to extracellular H_2_O_2_, cells were acutely exposed to either low (10 µM) or high (300 µM) concentrations of exogenous H_2_O_2_. Additionally, menadione (5 μM), a polycyclic aromatic ketone, was included to acutely generate intracellular superoxide anions (O_2_^●−^) by redox-cycling [32]. 

Untargeted metabolomics revealed that the metabolic response to the different ROS exposures differs considerably (Supplementary Table S1). DAO-derived H_2_O_2_ induced only a subtle change in identified metabolites (a total of 39 metabolites significantly changed) over the course of time. Menadione (5 µM) and low extracellular H_2_O_2_ (10 µM) showed a similar effect with a total of 93 vs. 73 metabolites significantly changed. For both, metabolic changes peaked at 270 min. Major metabolic changes were observed in response to the high concentration of H_2_O_2_ (300 µM), leading to a total of 358 metabolites changed. Altered metabolites included those involved in redox homeostasis, energy, and nucleotide synthesis (Figure 2A,B, Supplementary Figure S3, Supplementary Table S2). These changes occurred as early as 3 min after the beginning of exposure, and reverted to baseline within 270 min. 

**Figure 3:**
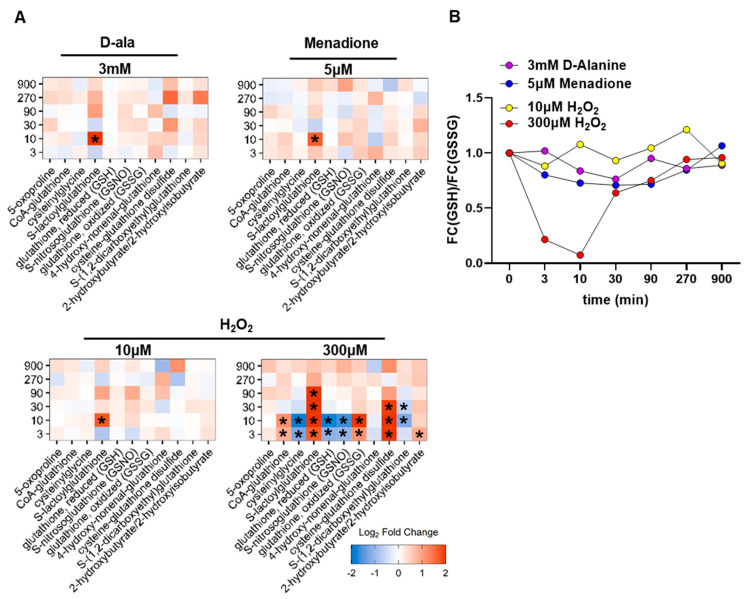
Changes in glutathione and glutathione-related metabolites in HUVEC upon exposure to different ROS. (**A**): Time course metabolic changes in glutathione metabolism. (**B**): Glutathione redox state in HUVEC over the course of time treatment with oxidative stimuli. FC = fold change. A&B: *n* = 3. * *p*-value < 0.05.

The glutathione metabolism is an essential and central antioxidant pathway. We therefore analyzed how this pathway is affected by the different oxidants (Figure 3A). High H_2_O_2_ had the strongest effect on glutathione metabolism, leading to a significant increase in oxidized glutathione species such as cysteine-glutathione disulfide. Furthermore, glutathione exhibited a transient decrease in the abundance of its reduced state in response to 300 µM H_2_O_2_ that recovered within 30 min. Unexpectedly, reduced glutathione was not affected by either DAO-derived H_2_O_2_ or menadione (Figure 3B). Nevertheless, all types of ROS significantly increased S-lactoylglutathione after 10 min. This metabolite is an intermediate in the detoxification of methylglyoxal, which is generated as a side product of upper glycolysis. Methylglyoxal is detoxified by the glyoxalase system, a composition of two enzymes, in which the first enzyme (glyoxalase I) converts the hemithioacetal that forms spontaneously between methylglyoxal and GSH to S-D-lactoylglutathione. The second enzyme (glyoxalase II) converts S-D-lactoylglutathione to GSH and D-lactate (Supplementary Figure S2C). An increase in methyglyoxal can occur under stress situations such as elevated ROS formation [33]. Methylglyoxal itself is highly reactive and therefore cannot be detected with the analytic methods used here. A close look at glycolysis revealed a general increase in metabolites of the upper glycolysis pathway (e.g., dihydroxyacetone phosphate, DHAP) and a decrease in metabolites of the lower glycolysis pathway (e.g., phosphoenolpyruvate, PEP) in response to exposure to H_2_O_2_ (300 µM). This effect was, however, not observed after exposure to DAO-derived H_2_O_2_ or menadione (Supplementary Figure S2A). Recent studies demonstrated that a short-term antioxidant response can be mediated by redox-sensitive enzymes in the lower glycolysis pathway. High H_2_O_2_ concentration inhibits glyceraldehyde 3-phosphate dehydrogenase (GAPDH) by promoting the formation of an intermolecular disulfide bond [34,35]. Therefore, an accumulation of DHAP is noticeable after exposure to high H_2_O_2_. The increase in DHAP is consistent with the accumulation of S-lactoylglutathione as methyglyoxal is formed during the non-enzymatic phosphate elimination of DHAP (Supplementary Figure S2C). Additionally, an overall decrease of nucleoside triphosphates (ATP and GTP) and an increase of nucleoside monophosphates emphasizes the cellular needs during stress defense, which includes the generation of nucleotide precursors for repair of DNA (Supplementary Figure S2B, Supplementary Table S1). NADPH and NADP+ were not detected in the experiment. Notably, only extracellular H_2_O_2_ at high concentration led to major changes in the metabolism of HUVEC. Therefore, we assume that the site and the concentration of ROS are central to elicit metabolic changes.

### 3.3. ROS Lead to Different Gene Expression Responses by HUVEC

Cells invoke specialized gene programs to cope with stress. In order to identify whether the type, concentration, and site (intra- vs. extracellular) of ROS exposure elicit differential gene expression, MACEseq was performed.

**Figure 4:**
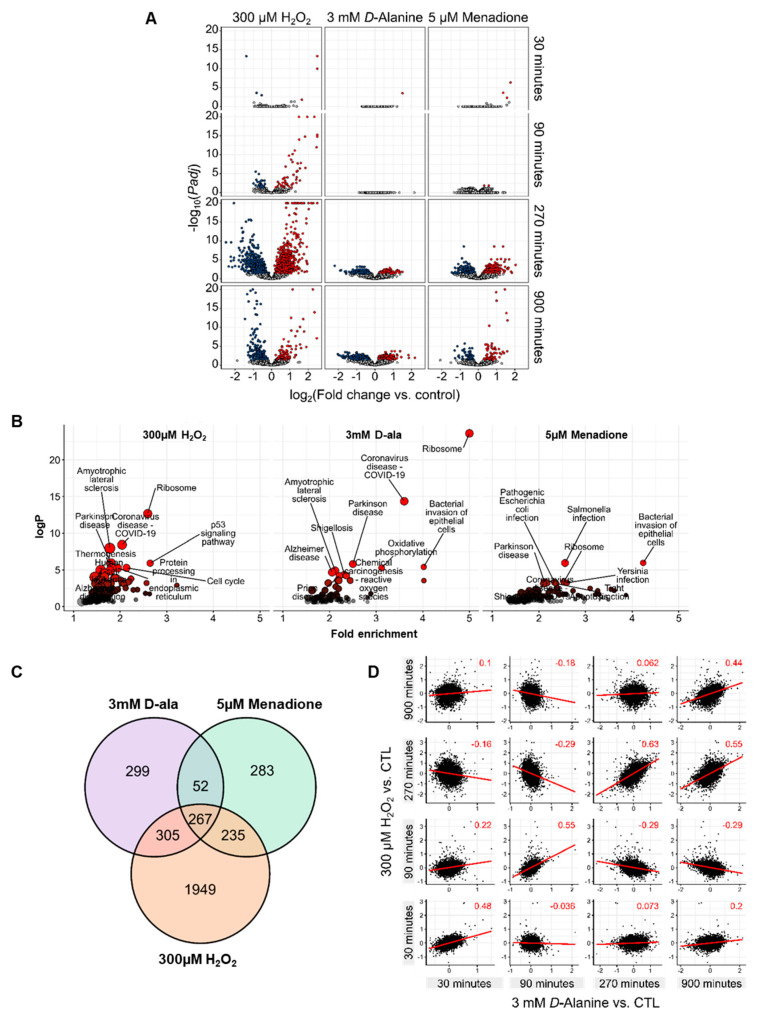
Different ROS lead to a different time-dependent transcriptomic response of HUVEC. (**A**): Differentially expressed genes after stimulation with 300 μM H_2_O_2_, 3 mM D-alanine or 5 μM menadione over the course of 30, 90, 270 and 900 min (significantly changed genes (p_adj_ < 0.05) are highlighted in blue and red). (**B**): Pathway annotation of significantly altered genes in A. (**C**): Venn diagram of significantly regulated genes in the treatments as indicated. (**D**): Correlation analysis for significantly DEGs comparing exogenous versus DAO-derived H_2_O_2_.

Similar to the metabolic measurements, exposure to 300 µM H_2_O_2_ induced the most pronounced transcriptomic changes (3540 DEGs, differentially expressed genes) in comparison to the other treatments (3 mM D-alanine 1575 DEGs; menadione 1236 DEGs) (Figure 4A, Supplementary Figure S3, Supplementary Table S3). Ten µM H_2_O_2_ led to no significant differences in gene expression as compared to untreated HUVEC. Thus, we focused our subsequent analysis on 300 µM H_2_O_2,_ 3 mM D-alanine and 5 µM menadione.

With respect to the temporal changes in gene expression, 300 μM extracellular H_2_O_2_ induced an early gene response 30 min after exposure, while most changes in gene expression occurred at 270 min (Figure 4A). In contrast, changes caused by 3 mM D-alanine and 5 µM menadione appeared only after 270 min.

To gain a comprehensive picture of the molecular pathways affected by exposure to different ROS, a pathway enrichment analysis using the Kyoto Encyclopedia of Genes and Genome (KEGG) database considering the significantly (p_adj_ < 0.05) changed genes for all time points was performed. High H_2_O_2_ concentration (300 µM) significantly induced genes of the p53 signaling pathway and cell cycle-related genes. Stress-responsive genes such as the cell cycle regulator GADD45A (Growth Arrest and DNA Damage Inducible Alpha) and PHLDA3 (Pleckstrin Homology Like Domain Family A Member 3) were increased after exposure to high concentration of H_2_O_2_. Moreover, induction of CDKN1A/p21 (Cyclin Dependent Kinase Inhibitor 1A) suggests that H_2_O_2_ promotes a senescent phenotype in EC (Supplementary Figure S4A, Supplementary Table S2). This is consistent with the known effect of H_2_O_2_ for stress-induced cell cycle arrest [36]. This effect did not occur after exposure to menadione or stimulation with D-alanine. Nevertheless, all oxidants significantly changed ribosome-associated RNAs (rRNA). rRNAs are bound to ribosomal proteins to form small and large ribosome subunits. They can be chemically modified by ROS, which we assume leads to an upregulation of the rRNA transcripts necessary to maintain ribosomal functionality (Figure 4B, Supplementary Figure S4A). We further described the similarities between high concentration of H_2_O_2_, DAO-derived H_2_O_2,_ and exposure to menadione. A Venn diagram shows that 267 genes were changed by all oxidative stimuli (Figure 4C). 

To gain insights into their regulation, we checked whether these genes are under control of similar transcription factors. Transcription Factor Enrichment Analysis (TFEA) identified 34 transcription factors (TF) such as TAF1, TAF7, MYC, ATF2, and YY1 as putative regulators of the DEGs common to all treatments (Supplementary Figure S4B). These TF (among others) highly regulate ribosomal-associated genes that are, in fact, enriched in all applied oxidative stimuli. Last, we investigated whether intracellular H_2_O_2_ induces any similar transcriptional response to exogenous H_2_O_2_ (300 µM) in HUVEC. For this, log fold change correlation analysis of the differentially expressed genes for each individual time point was performed. As shown in Figure 4D, a positive correlation in gene expression exists for exposure to H_2_O_2_ (300 µM) and DAO-derived H_2_O_2_ (3 mM D-alanine). This suggests that, despite the fact that H_2_O_2_ induces the largest changes in gene expression, a positive correlation exists for genes at equal time points, independent of the source of H_2_O_2_. 

Altogether, the results show that only exogenous H_2_O_2_ at high concentrations induces the classical stress-induced senescence markers (e.g., p21). Both menadione and DAO-derived H_2_O_2_ did not elicit this effect. Nevertheless, all treatments induce genes linked to ribosomal function while a positive correlation occurs in gene expression changes between high extracellular H_2_O_2_ and DAO-derived H_2_O_2_.

### 3.4. Only 300 µM H_2_O_2_ Overoxidizes Peroxiredoxins in HUVEC

As the major changes in metabolomics and transcriptomics of HUVEC were almost exclusively induced by exogenous H_2_O_2_ at high concentrations (300 µM), it appears that the type, the concentration, and the localization determine the biological effect of ROS exposure. Therefore, we took a close look at redox sensor proteins to detect disturbances in redox homeostasis. As thiol-specific antioxidants, peroxiredoxins (Prx) act as redox sensors. These enzymes harbor a peroxidatic cysteine that can be directly oxidized to protect cellular components from oxidative damage. Upon oxidation, Prx can form dimers or multimers with other Prx or proteins. Furthermore, the redox-sensitive cysteine of Prx can be irreversibly overoxidized to a sulfonic acid (RSO_3_^−^). Importantly, oxidized Prx are reduced by the thioredoxin system, which utilizes NADPH as reducing partner. As the oxidative status of Prx is a robust readout of the oxidation level of a cell [7,37,38], we investigated how exogenous and DAO-derived H_2_O_2_ as well as menadione oxidize Prx. 

**Figure 5:**
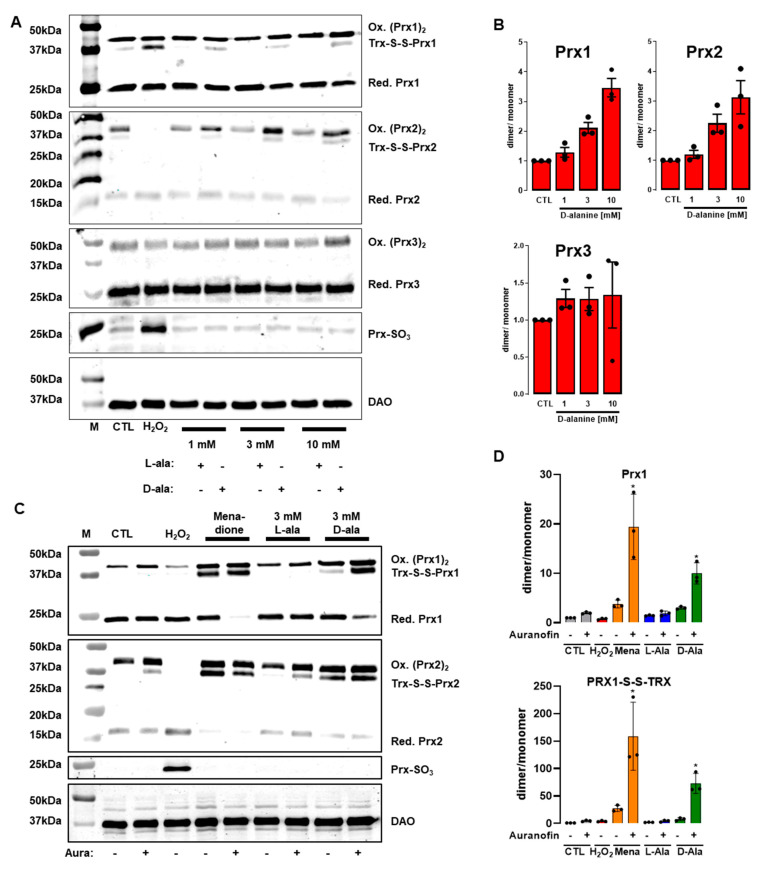
Oxidation of peroxiredoxins (Prx) in response to exogenous or DAO-derived H_2_O_2_ or menadione. (**A**): Representative redox western blot (30 µg protein) for Prx1, Prx2, Prx3, and Prx-SO_3_ after stimulation with different concentration of D- or L-Ala in HUVEC. (**B**): Quantification of redox-western blots by densitometry (*n* = 3). (**C**): Redox western blot and quantification (**D**) for Prx1 and Prx2 with HUVEC-DAO pre-incubated with auranofin (20 min, 3 µM) prior D- or L-ala stimulation, * *p* < 0.05 as compared to CTL with auranofin. One-way-ANOVA with Bonferroni correction.

Addition of D-alanine led to a dose-dependent increase in Prx-dimerization in HUVEC-DAO with a pronounced effect on the Prx1 and Prx2 isoenzymes but not in Prx3 (Figure 5A,B). The increase in Prx-dimerization was accompanied by a decrease in the corresponding Prx monomer. This effect on Prx1 and Prx2 dimerization was also detectable in HEK293 cells that overexpress DAO (HEK-DAO). Unlike the case with HUVEC, Prx3 was oxidized upon addition of 3 mM and 10 mM of D-ala in HEK-DAO. Furthermore, DAO-derived H_2_O_2_ led to the formation of Prx4 multimers in HEK-DAO (Supplementary Figure S5).

Stimulation of HUVEC-DAO with D-alanine increased the formation of the thioredoxin-S-S-peroxiredoxin dimer (Figure 5A). Remarkably, D-alanine did not lead to overoxidation of peroxiredoxins (Prx-SO_3_), which was only induced by 300 µM exogenous H_2_O_2_ (Figure 5A,B). We therefore speculated that Prx-SO_3_ only accumulated in response to intracellular H_2_O_2_ if the thioredoxin reductase system is inhibited. To test this, HUVEC were pre-incubated with auranofin (20 min, 3 µM) prior to addition of D-alanine. Blocking thioredoxin reductase increased the ratio of dimer to monomer of Prx1 in control cells by two-fold whereas this ratio for Prx-1-S-S-thioredoxin increased by six-fold (Figure 5C,D). Contrary to our expectation, pre-incubation with auranofin did not facilitate the accumulation of Prx-SO_3_ in response to menadione or DAO-derived H_2_O_2_ (Figure 5C). However, the presence of auranofin, menadione and DAO-derived H_2_O_2_ caused a massive increase in Prx-1 dimer as well as Prx-1-S-S-thioredoxin. Thus, the localization and concentration of ROS exposure are important factors when considering their effect on the redox-status of a cell. While DAO-derived H_2_O_2_ and menadione-derived superoxide are generated intracellularly, the antioxidant system takes effect immediately. Only high amounts of H_2_O_2_ added to the extracellular space might overcome the cellular antioxidant capacity and lead to Prx-overoxidation.

## 4. Discussion

In this study we compared how different types and concentrations of ROS modulate the metabolic and transcriptomic response of HUVEC over the course of time. To generate H_2_O_2_ intracellularly, we utilized a chemogenetic approach with DAO. DAO contains a peroxisomal targeting sequence (PTS), but immunofluorescence showed that the enzyme, in this overexpression system, is distributed throughout the whole cell. Therefore, we did not genetically manipulate its subcellular localization by removing the PTS or addition of a different subcellular target sequence. Interestingly, the DAO approach was used previously in an endothelial cell line (EA.hy926) to study how the enzyme targeted different subcellular compartments modulates endothelial cell phosphorylation pathways. DAO-derived H_2_O_2_ mediates eNOS phosphorylation via AMPK activation when the enzyme is directed to the nucleus by an importing sequence. Cytosolic or caveolae-targeted DAO had no impact on eNOS phosphorylation [13,39]. Furthermore in another study, DAO was overexpressed in the heart of mice and its activation, by feeding the mice D-alanine, which led to cardiac dysfunction [18]. 

Nevertheless, the DAO approach has not yet been explored in an untargeted metabolomics and transcriptomics study. In fact, there are only few studies that describe the short-term and long-term metabolic effects of exposure to H_2_O_2_. In yeast and many human cell types, it was shown that inhibition of glycolytic enzymes by H_2_O_2_ leads to an accumulation of glycolytic intermediates that consequently induce an increased flux into the pentose phosphate pathway (PPP), through both the oxidative and non-oxidative branches [40,41,42,43]. This matches our findings and is compatible with the previously shown oxidative inhibition of GAPDH. Kuehne et al. (2015) directly showed an upregulation of the PPP in human skin fibroblasts as a first line response after exposure to 500 µM H_2_O_2_. By using ultra-short ^13^C labelling experiments, the authors provided evidence for multiple cycling of carbon backbones in the oxidative PPP, potentially maximizing NADPH reduction. Hence, NADPH is required as a reducing equivalent, which maintains the active form of catalase and is a cofactor of TRX and GSH reductase [35,44]. Additionally, it has been shown that quiescent EC, in a manner different from that of proliferating EC, protect themselves against oxidative stress by increasing fatty acid oxidation up to three-fold to generate NADPH via isocitrate dehydrogenase and malic enzyme [45]. Furthermore, H_2_O_2_ affected phosphorylated nucleotides and produced a reduction in ATP and GTP, which was paralleled by an increase in their respective mono-phosphorylated forms [46]. It was quite surprising to observe that only H_2_O_2_ at high concentration induced most metabolic changes in HUVEC. As early as 3 min after the addition of high concentration of H_2_O_2,_ significant changes in the antioxidant system became apparent. A profound reduction in the GSH/GSSG ratio was observed after 10 min and this ratio was back to the basal situation after 270 min, pointing to a transient effect. High concentration of H_2_O_2_ also increased the concentration of methionine sulfoxide and reduced the concentration of isocitrate and other TCA cycle metabolites. The later effect was also observed in menadione-treated HUVEC and is known to be linked to an inhibition of aconitase via oxidation of an Fe−S cluster [9]. The only common metabolite that we were able to detect altered in all treatments at the 10 min time point was S-lactoylglutathione. This metabolite is formed upon reaction of glutathione with methylglyoxal [33]. An increase in methylglyoxal can occur under stress conditions likely due to several events such as the reversible S-glutathionylation and inhibition of GAPDH [47]. Methylglyoxal itself was not detected in the experiments, likely due to its high reactivity, but considering the fact that endothelial cells are highly glycolytic, an efficient removal of methylglyoxal in GSH-dependent manner is essential. 

The changes in gene expression observed in HUVEC were mainly caused by exposure to 300 µM H_2_O_2_ that activated, among others, the p53 pathway. It is accepted that low to moderate levels of ROS activate p53-related genes that increase the time needed for cell repair (e.g., cell cycle arrest and autophagy). With higher levels of ROS, p53 facilitates cellular stress and induces apoptosis to prevent aberrant cell proliferation. Exposure to menadione induces selective increases in genes of the p53 pathway, suggesting that the intensity and location of the oxidation are important parameters that would determine the function of p53 in regulating the signaling outcome [48]. All oxidative stimuli had a significant impact on ribosome-associated RNA. Both ribosomal RNA and ribosomal proteins can be chemically modified by ROS, leading to a loss of their function. Ribosomal RNA is the structural and functional core of the ribosome. ROS can chemically modify the base and sugar moieties of rRNA, generating a basic site and strand breaks. Among proteins of the translational machinery, the impact of oxidative stress on the ribosome remains the least studied [49,50]. Due to its high cellular abundance, RNA is more frequently subject to oxidative damage in comparison to DNA [49]. Transcription factor analysis revealed ATF2, MYC, and TAF1/7 related genes commonly changed among the applied redox stimuli. ATF2 and MYC sense cellular stress caused by increased ROS concentrations. They regulate DNA damage response and cell cycle progression [51,52]. Moreover, TAF-related genes are required for basal transcription. Interestingly, all TFs regulate the expression of ribosomal genes, e.g., RPL4, which encodes the 60 S subunit of the ribosome [53]. Indeed, the findings of this study suggest that EC upregulate ribosomal RNAs as a response to different redox stimuli. This implies that rRNA is a primary target for oxidative stress in EC and this phenomenon has not been extensively studied in EC and can be the subject to future research. 

High extracellular concentration of H_2_O_2_ had a stronger impact on gene expression and metabolism compared to menadione and DAO-derived H_2_O_2_. Thus, we analysed the oxidation status of Prx enzymes. Only H_2_O_2_ at high concentrations induced an overoxidation of Prx (Prx-SO_3_). Blocking thioredoxin reductases with auranofin did not induce the formation of Prx-SO_3_ in D-alanine treated cells. This points to a high intracellular reducing capacity of EC and the relevance of compartmentalized ROS production for signalling. The *floodgate* model suggests that scavenging enzymes such as Prx must be inactivated, so H_2_O_2_ can directly oxidize target proteins. The irreversible inactivation of Prx by overoxidation allows endogenous H_2_O_2_ concentrations to build up, which promotes redox signalling by direct oxidation of target proteins and other biomolecules. Others have shown that reaction of H_2_O_2_ with thiols is too slow to outcompete peroxiredoxins to exert a direct redox signaling [54]. In fact, peroxiredoxins indirectly facilitate H_2_O_2_ sensing and oxidize target proteins [55,56] in a redox-relay model, transferring oxidizing equivalents to the transcription factor STAT3 or to the kinases ASK1 and MEKK4 [56,57,58,59]. 

As shown here, there is a substantial difference in the metabolic and transcriptomic response by EC to extracellular H_2_O_2_ vs. intracellular H_2_O_2_. This is consistent with increasing recognition of subcellular compartmentalization of redox processes. Except for the endoplasmic reticulum, intracellular redox potentials are largely more reducing in comparison to the extracellular space. In particular, the extracellular space contains recognizable amounts of reactive intermediates and several available protein targets. Thiol-redox regulation of the extracellular space is associated with cellular signaling cascades. Indeed, the pathways that respond to ROS are essentially the extracellular signal-regulated kinases (ERK1/2), c-Jun NH2-terminal kinases (JNKs), and p38 kinase. The ERKs family can be activated by growth factors as a response to oxidative stress. Vascular smooth muscle cells (VSMC) secrete cyclophilin A, a member of the immunophilin family, in response to oxidative stress, which mediates ERK1/2 activation. The JNKs and p38 kinases are primarily involved in the cellular stress condition and are activated by extracellular H_2_O_2_ in smooth muscle cells [60,61]. 

## 5. Conclusions

In summary, only high concentrations (300 µM) of extracellular H_2_O_2_ induced significant changes in metabolic pathways of redox homeostasis, energy production, and nucleotide synthesis. Likewise, high concentration of H_2_O_2_ caused major changes in gene expression and oxidation of peroxiredoxin enzymes. Collectively, these findings suggest that the source and the concentration of ROS are important to elicit changes in metabolism and gene expression. Our data indicate that EC have sufficient intracellular reducing capacity to scavenge intracellularly produced H_2_O_2_. This is a possible explanation for the different effects of intracellular DAO-derived H_2_O_2_ and menadione-derived superoxide in comparison to high exogenous H_2_O_2_.

## Data Availability

Data is contained within the article and supplementary material.

## Supplementary Materials

The following supporting information can be downloaded at: https://www.mdpi.com/article/10.3390/antiox11020434/s1. Figure S1: H_2_O_2_ production by HUVEC-DAO; Figure S2: Changes in glycolysis and nucleotide metabolism in HUVEC in response to different oxidative stimuli; Figure S3: High degree of dissimilarity between effects of different ROS exposures to HUVEC; Figure S4: Differentially expressed genes in HUVEC in response to different ROS; Figure S5: DAO-derived H_2_O_2_ results in a dose-dependent oxidation of peroxiredoxins in HEK-DAO; Table S1: Summary of altered metabolites; Table S2: Metabolomics of HUVEC (excel file); Table S3: RNAseq by MACE: all DEG (excel file).
