# Supplementary material for: Reactive Oxygen Species Differentially Modulate the Metabolic and Transcriptomic Response of Endothelial Cells
Source: Antioxidants (Basel). 2022 Feb 21;11(2):434. doi: 10.3390/antiox11020434 (PMC8869421; doi:10.3390/antiox11020434)
Supplement: Supplementary file 1 [file antioxidants-11-00434-s001.zip › antioxidants-1602308-SM-final.pdf]

Supplementary Materials: Reactive Oxygen Species Differentially Modulate the Metabolic and Transcriptomic Response of Endothelial Cells

Niklas Müller [1,2], Timothy Warwick [1,2], Kurt Noack [1,2], Pedro F. Malacarne [1,2], Arthur J.L. Cooper [3], Norbert Weissmann [4], Katrin Schröder [1,2], Ralf P. Brandes [1,2] and Flávia Rezende [1,2,*]

[1] Institute for Cardiovascular Physiology, Goethe University, Theodor-Stern Kai 7, 60590 Frankfurt, Germany; nmueller@vrc.uni-frankfurt.de (K.M.); warwick@vrc.uni-frankfurt.de (W.T.); kunoack@students.uni-mainz.de (K.N.); malacarne@vrc.uni-frankfurt.de (P.F.M.); schroeder@vrc.uni-frankfurt.de (K.S.); brandes@vrc.uni-frankfurt.de (R.P.B.)

[2] German Center of Cardiovascular Research (DZHK), Partner Site Rhein Main, 60590 Frankfurt, Germany

[3] Department of Biochemistry and Molecular Biology, New York Medical College, 15 Dana Road, Valhalla, NY 10595, USA; arthur_cooper@nymc.edu

[4] Excellence Cluster Cardio-Pulmonary Institute (CPI), University of Giessen and Marburg Lung Center (UGMLC), Member of the German Center for Lung Research (DZL), Justus-Liebig-University, Giessen, 35390 Germany; norbert.weissmann@innere.med.uni-giessen.de

* Correspondence: rezende@vrc.uni-frankfurt.de; Tel.: +49-69-6301-85321; Fax: +49-69-6301-7668

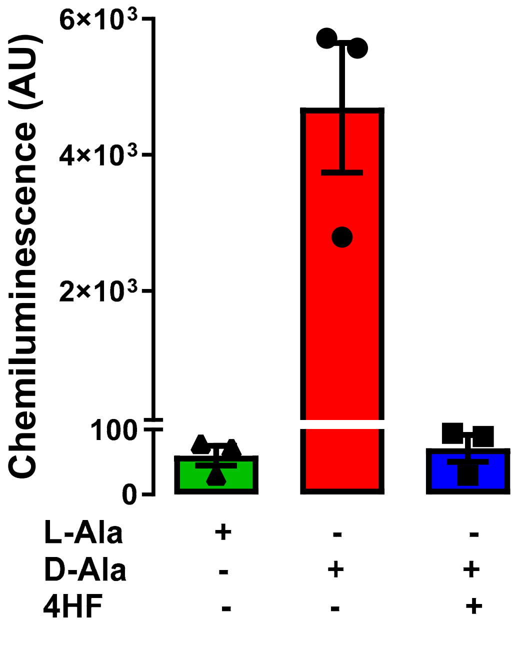

Supplementary Figure S1: **H_2O_2 production by HUVEC-DAO.** H_2O_2 (n=3) assessed by chemiluminescence with luminol (100 µM)/ HRP (1 U/mL) after exposure to D- or L-alanine with or without pre-incubation (10 min) with the DAO inhibitor 4HF (1 µmol/L) (n=3).

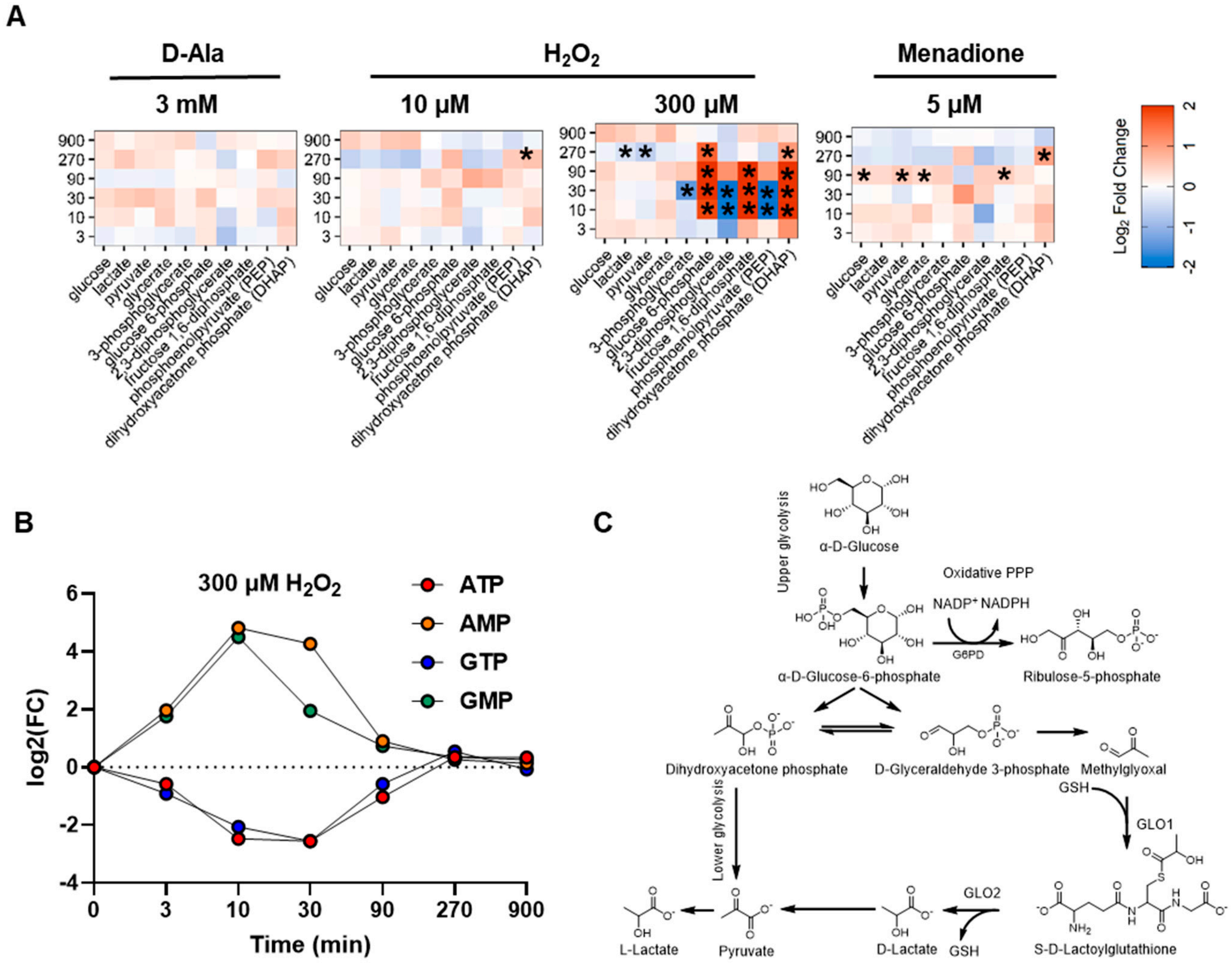

Supplementary Figure S2: Changes in glycolysis and nucleotide metabolism in HUVEC in response to different oxidative stimuli. (**A**): Heat maps of glucose metabolism. (**B**): Changes in ATP, AMP, GTP and GMP after exposure to 300 µM H_2O_2. (**C**): Fates of glucose and S-lactoylglutathione pathway.

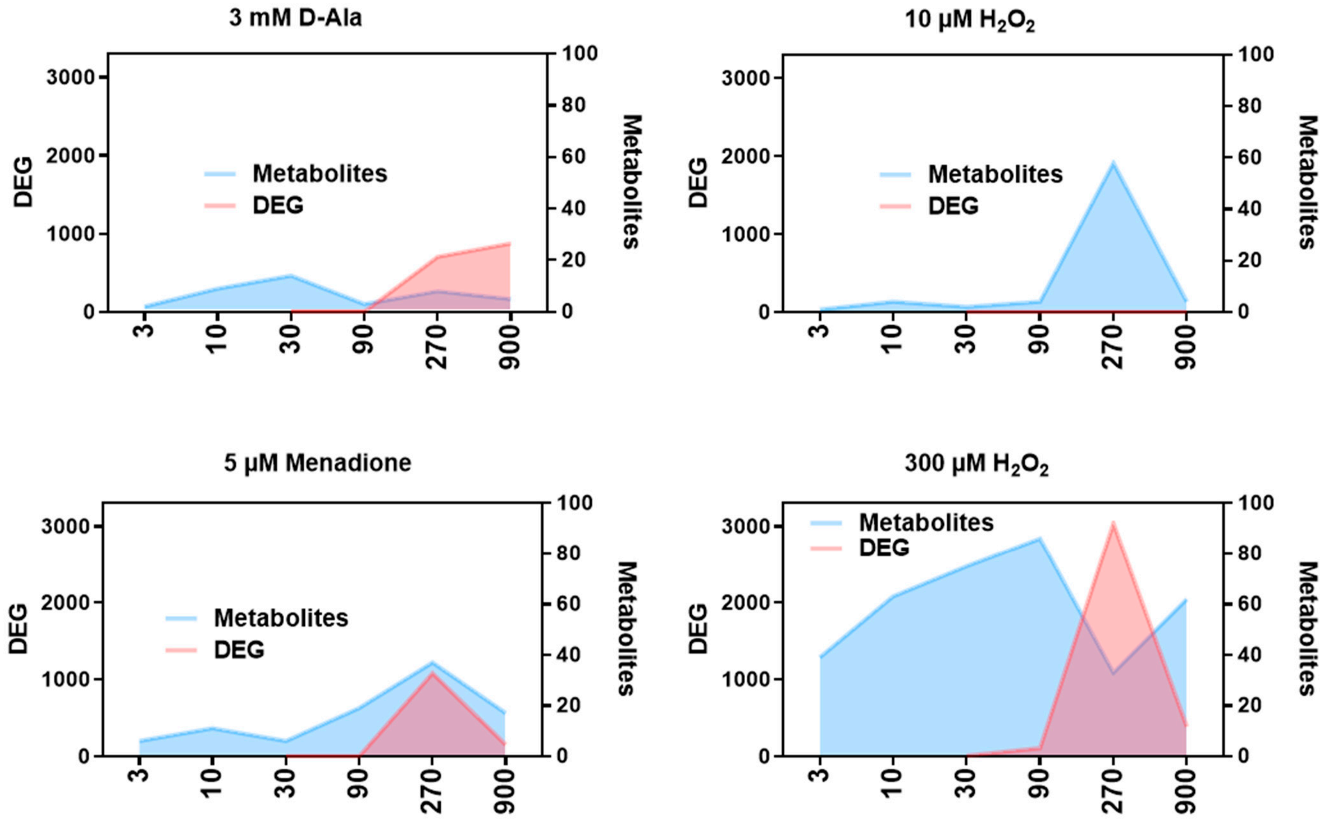

Supplementary Figure S3: High degree of dissimilarity between effects of different ROS exposures to HUVEC. Time course changes in metabolomics and transcriptomics of HUVEC in response to different oxidative stimuli.

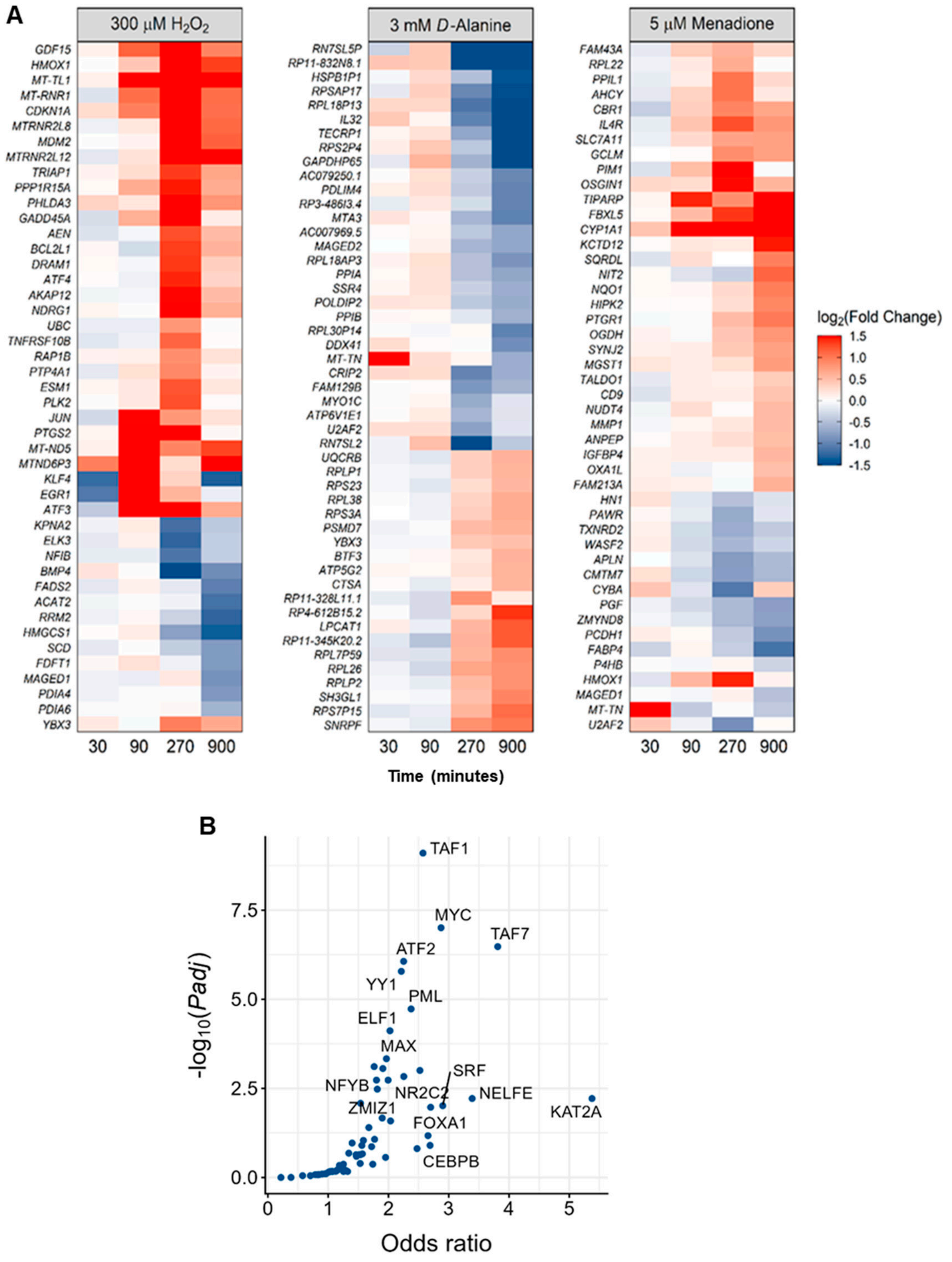

Supplementary Figure S4: Differentially expressed genes in HUVEC in response to different ROS. (**A**): Heatmap of DEGs. (**B**): Transcription factor analysis performed with ENCODE and CheA for the genes commonly regulated by 300 μM H_2O_2, 3 mM D-Ala and 5 μM menadione.

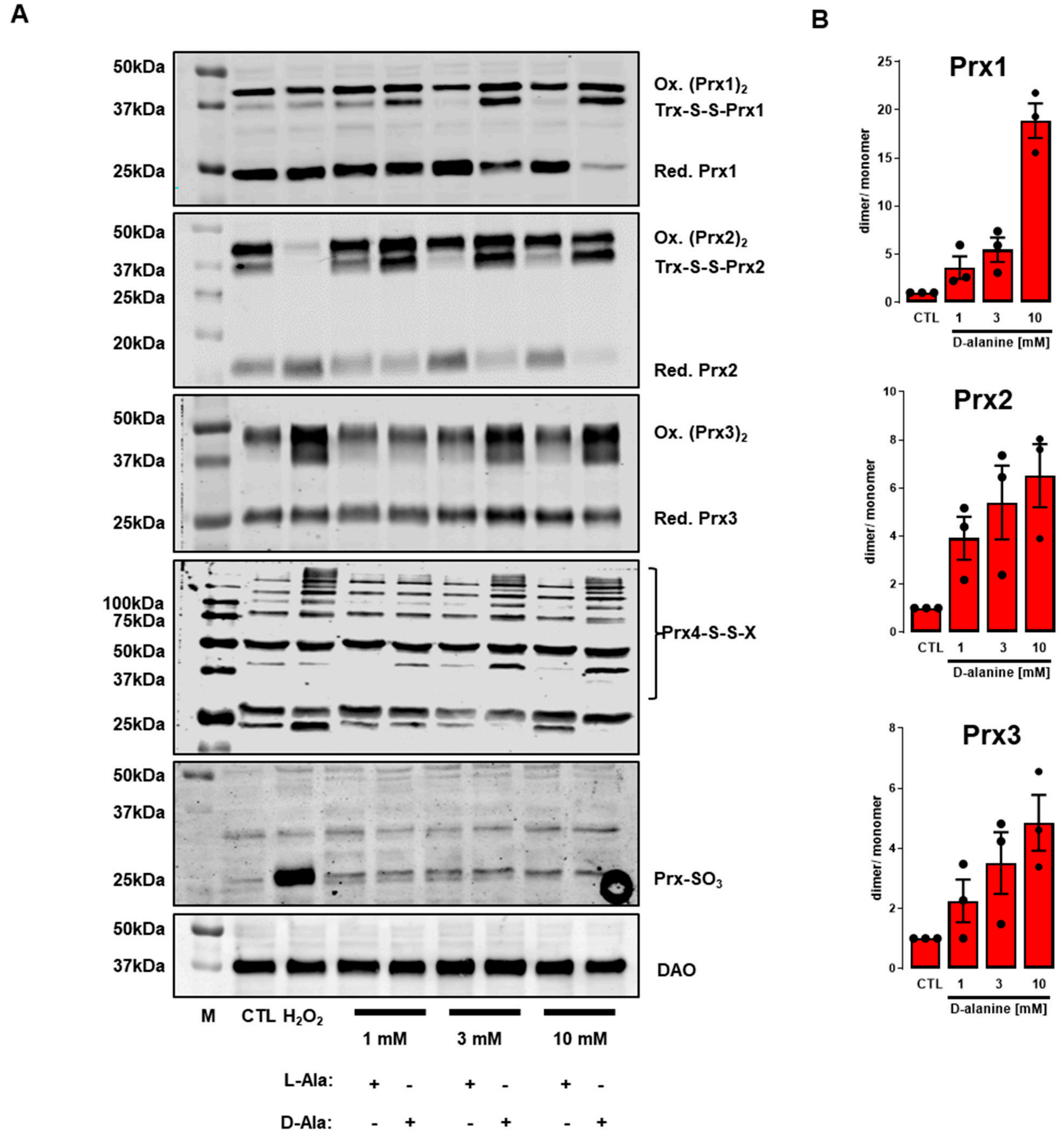

Supplementary Figure S5: DAO-derived H_2O_2 results in a dose-dependent oxidation of peroxiredoxins in HEK-DAO. (**A**): Representative redox western blot for Prx1, Prx2, Prx3, Prx4 and Prx-SO_3 after exposure to different concentration of D- or L-Ala. (**B**): Quantification of redox western blotting by densitometry (n=3).

	3 mM D-Ala/CTL					
	3′	10′	30′	90′	270′	900′
↑	1	9	14	1	6	3
↓	1	0		2	2	2
**Total**	2	9	14	3	8	5

	10 µM H_2O_2/CTL						300 µM H_2O_2/CTL					
	3′	10′	30′	90′	270′	900′	3′	10′	30′	90′	270′	900′
↑	0	4	1	4	2	3	23	39	44	56	23	60
↓	1	0	1	0	56	1	16	24	31	30	10	2
**Total**	1	4	2	4	58	4	39	63	75	86	33	62

	5 µM Menadione/CTL					
	3′	10′	30′	90′	270′	900′
↑	1	6	3	16	18	4
↓	5	5	3	3	19	13
**Total**	6	11	6	19	37	17

Supplementary Table S1: Summary of altered metabolites.
